# Ethnic- and gender-specific differences in the prevalence of HIV among patients in opioid maintenance treatment—a case register analysis

**DOI:** 10.1186/1477-7517-11-23

**Published:** 2014-08-18

**Authors:** Michael Liebrenz, Rudolf Stohler, Carlos Nordt

**Affiliations:** 1 Research Group on Substance Use Disorders, Psychiatric University Hospital, Selnaustrasse 9, 8001 Zurich, Switzerland; 2 Department of Psychiatry, New York State Psychiatric Institute, Columbia University Medical Center, 1051 Riverside Drive, New York, NY 10032, USA

## Abstract

**Background:**

We have sought to identify ethnic- and gender-specific differences in HIV prevalence among heroin users receiving opioid maintenance treatment in the canton of Zurich, Switzerland.

**Methods:**

We used a generalized linear model (GEE) to analyze data from the anonymized case register for all opioid maintenance treatments in the canton of Zurich. Patients who received either methadone or buprenorphine between 1991 and 2012 (*n* = 11,422) were evaluated for gender (male vs. female), ethnic background (Swiss vs. non-Swiss), and lifetime method of drug use (ever injector vs. non-injector). We addressed missing data by multiple imputation.

**Results:**

The overall prevalence of HIV among patients declined substantially from 33.7% in 1991 to 10.6% in 2012 in the complete dataset. In the imputed datasets, the respective prevalence dropped from 32.8% in 1991 to 9.7% in 2012. Non-injectors had a four to five times lower risk ratio (RR) compared to the reference group, ‘Swiss males who ever injected’. In addition, we found a significantly higher risk ratio of HIV prevalence among females who had ever injected; this was true both for the complete dataset and the imputed dataset (Swiss RR 1.18 CI 95% 1.04–1.34, non-Swiss RR 1.58 CI 95% 1.18–2.12).

**Conclusion:**

In this population, gender, ethnic background, and lifetime method of drug use influenced the risk of being HIV positive. Different access to treatment and different characteristics of risk exposure among certain subgroups might explain these findings. In particular, the higher risk for women who inject drugs—especially for those with an immigrant background—warrants additional research. Further exploration should identify what factors deter women from using available HIV-prevention measures and whether and how these measures can be better adapted to high-risk groups.

## Background

### Methadone as HIV prevention

Opioid maintenance treatment (OMT) with methadone or buprenorphine is widely recognized as an effective HIV-prevention intervention for patients with an opioid dependence [1-5]. It has proved beneficial for injection drug users (IDU) and non-injection drug users (non-IDU) alike and is associated with a reduction in the incidence of HIV [6]. Patients in OMT use significantly less street opiates and in the case of IDUs, reduce the frequency of opiate injection and needle sharing [7-9]. The association between injection drug use and increased risk for acquiring HIV has been well documented for over 20 years [10], and it is clearly understood today that OMT reduces injection-related HIV risk behaviors [1,10,11]. However, OMTs’ usefulness is not limited to IDUs [1]: Multiple studies have shown that besides sexual contact with an IDU, alcohol and non-injection drug use represent significant risk factors for the acquisition of a new HIV infection in both genders, regardless of sexual orientation [6,12-14]. Among opioid-dependent patients trading sex for drugs, low-threshold access to OMT is known to significantly reduce engagement in prostitution and with it to decrease total numbers of sex partners and rates of unprotected sex [15,16]. Because of its vast health benefits, OMT is considered a primary and secondary HIV/AIDS prevention strategy; accordingly, methadone was included in the WHO list of essential drugs in 2005 [17,18].

### Prevalence of HIV among injection drug users

Data from the Swiss Federal Office of Public Health (SFOPH) indicate that the introduction of harm-reduction policies such as needle and syringe programs (NSP), as well as supervised consumption rooms (SCR) and the availability of OMT, resulted in a sharp decrease in the incidence of new HIV infection among IDUs: from more than 900 new infections in 1989 to 28 new positive tests in 2008 [19-22].

The prevalence of HIV in this subgroup has been reported to be fairly stable: between 5% for IDUs in a treatment setting and 10% in low-threshold facilities (LTF), such as needle and syringe programs or drug consumption facilities [23]. These data resulted from cross-sectional surveys conducted in 1993, 1994, 1996, 2000, and 2006 by means of questionnaires distributed to IDUs in LTFs in ten different cantons in Switzerland [24]. It is thereby estimated that over half of IDUs frequenting LTFs simultaneously receive OMT [23].

Additional data on the prevalence of IDU among HIV-infected patients stem from the Swiss HIV Cohort Study (SHCS), which originated in 1988 [25]. Since then, the study has continuously followed patients with HIV who undergo treatment at any of the five university hospitals in Switzerland, or in SHCS-associated regional hospitals or private doctors’ offices [26]. By March 2009, the SHCS covered a total of 15,624 patients, which is estimated to represent 45% of the cumulative number of HIV infections known to SFOPH and 69% of individuals with AIDS who reside in Switzerland. It was estimated that 29.5% of all ever-registered patients were infected through injection drug use and that 16.8% of those currently in treatment were similarly infected (data as of 2009) [26].

### Ethnicity, gender, substance use disorder, and HIV

Multiple studies have shown that immigration is often associated with increased substance use because of social difficulties and stressors [27] and that while patterns of substance use begin to resemble that of the native population over time [28], immigrants are initially more likely to engage in riskier injection behaviors and to share syringes [29,30]. Women with an immigration background and co-occurring substance use disorders are estimated to have an elevated risk of contracting HIV, either by injecting drugs, having sex with an injecting drug user, or through other high-risk sexual behaviors and absence of condom use [31,32]. Extensive literature shows that illegal immigrant women might be at increased risk of HIV either because of engaging in sex work or survival sex (e.g., exchanging sex for shelter, money, or other resources). This is due to the lack of employment [33-36].

Furthermore, studies from North America among White, African American, and Hispanic women participating in methadone maintenance suggest ethnic differences in substance use and sexual behaviors that increase the relative risk of HIV infection [31,37-39]. According to Grella et al. [39], Hispanic women more frequently stated familial influences, irregular condom use, and high-risk injection behavior. White women reported the highest levels of consistent condom use but were the least likely to use safer injection practices; and African-American women reported more use of alcohol and crack cocaine, both before and after entering OMT [39].

To our knowledge, however, epidemiological data on rates of correlation between HIV infection and gender and ethnicity in opioid maintenance treatment are not readily available, despite these cited findings.

### Immigrants in Switzerland and Zurich

Switzerland has one of the largest foreign populations but is not regarded by most natives as an immigration country [40]. In recent years, the population of Switzerland grew by approximately 80,000 immigrants per year, mostly due to the needs of Switzerland’s economy [41,42]. In January 2013, the canton of Zurich had 1,406,000 inhabitants. As of November 2013, the canton of Zurich had 356,753 permanent non-Swiss residents. Of these residents, 190,262 were men and 166,491 were women, 239,364 were citizens of an EU-28 or EFTA country, 9,676 were from Africa, 15,895 were from America (North, Central, and South), and 23,284 were from Asia [43]. Thus, the proportion of immigrants to Swiss nationals in the canton of Zurich was 25.4%. The number of illegal immigrants is relatively small. In 2005, it was estimated that about 20,000 inhabitants of the canton of Zurich had no legal documentation [44]. Our case, register includes all patients receiving OMT, regardless of their immigration status. It is mandatory for legal residents to carry health insurance (that covers OMT); if they do not have sufficient personal funds to buy it, the social welfare office steps in and covers the fees [45-47]. Illegal immigrants have access to the health care system, which requires all physicians to help those in need. Furthermore, public hospitals are required by law to provide treatment and not just emergency aid. Thus, access to the medical system for individuals without legal documentation is largely secured [48].

### Open questions

While data have shown that the introduction of harm-reduction policies in Switzerland significantly decreased the incidence of HIV and a stable prevalence of this virus in the general substance-using population, less is known about ethnic- and gender-specific differences in HIV status among heroin users currently receiving opioid maintenance treatment. The aim of our investigation was therefore to explore these issues by evaluating a comprehensive case register of maintenance treatments in the canton of Zurich. For the purpose of this investigation, we defined ‘immigrants’ as migrants to Europe—and more specifically to Switzerland.

## Methods

### Study area

Switzerland is a federal republic consisting of 26 cantons. It covers 15,940 square miles and has approximately 7.8 million inhabitants. Its most populous canton is Zurich, with 1.35 million residents, of whom 23.8% (321,000) are legal aliens [49].

In the mid-1990s, prevalence rates for opioid dependence were estimated at about 0.9% [50]. Since then, the Swiss Federal Office of Public Health and numerous other studies have reported a decrease in the prevalence of heroin use, which is estimated today to affect 18,500 to 25,000 individuals in Switzerland [19,23]. Still, approximately 25% of all heroin users live and die in the canton of Zurich [51].

### Database

Switzerland’s controlled substances legislation requires every initiation and termination of an opioid maintenance treatment to be registered with cantonal health authorities. Each of its 26 cantons is thereby obliged by law to maintain an independent database on treatment numbers [52,53]. Our research group has been accredited by the health authorities of the canton of Zurich to operate and evaluate its case register of maintenance treatments with methadone or buprenorphine. However, this mandate does not include the collection of data on heroin-assisted treatments.

Upon initiation of OMT, physicians are required to provide detailed but anonymous information about patients by completing a standard admission from. The questionnaire, which is also distributed at least twice annually (follow-up form) and at the end of treatment (discharge form), contains 24 questions on personal information (age, sex, nationality), previous substance use (drug of choice, frequency of consumption, type of application), and psychosocial characteristics (living conditions, educational level, employment status, interpersonal relationships), as well as former maintenance treatments and patients’ progress in current treatment (results of urinalyses, number of take-home doses). Special attention has been paid to document changes in the serological status of HIV, HBV, HCV, and vaccination status (HBV). On the admission form, physicians are asked to document whether a patient had been tested for HIV and to report the current status. However, answer choices include an option to keep this information confidential. These data are not solely collected for evaluation purposes—they are also used to call physicians’ attention to the possibility of infectious diseases in this high-risk group.

An individual code is assigned to each registration. This allows unequivocal identification of patients, following, for example, a change of treatment providers or an interruption of OMT. To ensure completeness of information, several measures are applied: The database software checks the appropriate range of data inputs and generates letters for providers who have failed to meet the requirements. Furthermore, prescriptions for methadone and buprenorphine issued by physicians in Zurich are controlled.

### Statistical analysis

The case register was established in 1991. From then until April 2013, we had registered 32,667 treatment episodes with 11,468 patients, which we defined as uninterrupted opioid maintenance treatments by the same provider using the same kind of opioid. Using data from admission forms, we obtained information about gender from almost all patients (except for 5.9%), if they were Swiss citizens or not (except for 12.8%) and if they had ever injected drugs (except for 7.7%). By using additional data from follow-up forms (which were distributed 6 months after admission for treatment) and data from discharge forms, we obtained HIV status on three out of four patients (except for 26.4%). As most patients had had several treatment episodes, we observed those whose lifetime injecting status changed over time (*n* = 1,369), as well as the few patients who had become HIV positive (*n* = 317). If available, we always applied the estimated year of status change to our analysis. Because we were missing data in regard to different variables, a complete dataset analysis could only utilize the information gathered from 65.9% of the total sample. We therefore applied the multiple imputation procedure implemented in the statistical software, SPSS 18.0.3, by using all variables of our model of interest (MOI) with fully conditional specification (FCS), including two-way interactions for nominal variables; and we set the number of imputed datasets to ten. We checked imputation for plausible values and made small corrections in fewer than eight cases of each imputed dataset, in order to maintain consistency.

For analysis, we produced a long dataset structure with one record per patient for each year, if he or she was in substitution treatment. For example, a patient whose first treatment episode began in 1992 and ended in 1994 and then had a second treatment episode of 2 months in 2000 was represented with four records in the long dataset (i.e., with the calendar year set to 1992, 1993, 1994, and 2000). Each record consisted of the following variables: a subject identifier, number of record of the subject, calendar year, year of birth, current age (=calendar year - year of birth), gender, nationality (Swiss or non-Swiss), current lifetime injecting status, and current HIV status.

As the overall proportion of HIV-positive patients in substitution treatment was greater than 10%, we modeled risk ratio (RR) instead of an odds ratio (OR), since the differential between the RR and OR increases with increasing rates. Therefore, we applied the extension of the modified Poisson regression model to prospective studies, with correlated binary data proposed recently by Zou and Donner [54]. The modified Poisson model was estimated by generalized estimating equation (GEE) method, using Poisson distribution and log as link function, and a repeated subject identifier for each patient, with an independent working correlation structure and robust estimator for covariance matrix, developed by Liang and Zeger [55]. The modified Poisson model does not have the convergence problems that commonly arise with log-binomial regression and is therefore a viable approach for estimation of risk ratio as an aggregated effect measure but is not a suitable procedure for predicting individual risk [54].

In order to find and present a parsimonious model, we rescaled year of birth and age with linear, squared, and cubic components that well fitted the observed data. Also, calendar year was rescaled and the logarithm taken. Note that the chosen rescaling and transformations do not have a clear interpretation itself as minor modifications of the used parameters would lead to similar GEE fits. As the parameter estimates of the GEE model are hard to interpret, we show figures giving the observed and model-fitted HIV prevalence in the complete and imputed dataset plotted by calendar year, year of birth, or age.

### Ethics

Zurich’s cantonal ethics committee approved the analysis of data.

## Results

Between 1991 and 1995, the annual number of patients in OMT with methadone or buprenorphine in the canton of Zurich increased from 2,015 to 3,736, remained almost stable until 2010, and then slightly declined in 2012 to 3,215 patients. During this period, we registered 11,422 patients, all of whom were included in the analysis using the imputation dataset, whereas the complete dataset analysis could use only the data from 7,557 patients due to missing data in relation to gender, nationality, and injecting or HIV status.

According to the first subject record in the long dataset, the group size of non-Swiss females who had never injected was *n* = 185 in the complete dataset as well as in the ten imputed datasets with mean size of 215.3 individuals, the smallest group (Table 1). Non-Swiss females who never had injected had also the lowest mean number of about five records in the long dataset as well as the lowest mean age of about 32 years compared to the other groups. The most sizable group was Swiss males who had ever injected (*N* = 2,733) in the complete dataset, as well as in the imputed datasets (mean of *n* = 3,192.2). This group had the highest mean age in the long dataset (36 years). Overall, apart from size, the groups did not strongly deviate with respect to age and number of records, in both the complete and the imputed datasets.

**Table 1:** Sample description using the long dataset (one record per patient for each year being in treatment)

	**Group size according to first subject record**		**Number of records (mean)**		**Age in years (mean; using all records)**	
	**Complete dataset**	**Imputed dataset**	**Complete dataset**	**Imputed dataset**	**Complete dataset**	**Imputed dataset**
Female, non-Swiss, non-injector	185	215.3	5.07	4.95	31.9	32.0
Female, non-Swiss, ever injector	188	225.5	5.71	5.20	34.2	33.9
Female, Swiss, non-injector	1,105	1,298.4	7.60	7.33	33.2	33.2
Female, Swiss, ever injector	1,505	1,738.2	7.26	6.81	34.0	33.9
Male, non-Swiss, non-injector	941	1,124.5	6.14	5.86	33.2	33.2
Male, non-Swiss, ever injector	830	984.9	5.25	4.96	34.8	34.7
Male, Swiss, non-injector	2,245	2,643.0	7.98	7.70	34.2	34.0
Male, Swiss, ever injector	2,733	3,192.2	7.30	6.94	36.0	35.9

**Figure 1:**
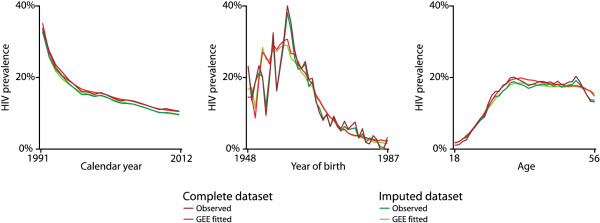
**HIV prevalence using all data from the complete and the imputed long dataset.** One record per patient for each year being in treatment. Plotted by calendar year, year of birth, or age, respectively.

**Figure 2:**
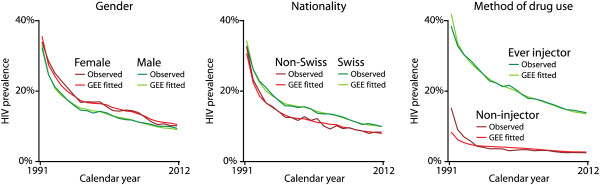
**HIV prevalence using all data from the imputed long dataset.** One record per patient for each year being in treatment. Plotted by calendar year, separate for gender, nationality and method of drug use.

Prevalence of HIV among all patients in OMT in the canton of Zurich has declined substantially over the course of the last 20 years. In 1991, the prevalence was 33.7%, whereas in 2012, it was only 10.6% in the complete dataset; in the imputed datasets, the respective prevalence dropped from 32.8% in 1991 to 9.7% in 2012. Our GEE model showed a quite complicated influence structure of age and year of birth, requiring linear, squared, and cubic components of both variables to fit the observed HIV prevalence. As these effects are hard to interpret from the numerical output (Table 2), we produced graphs showing the proportion of HIV prevalence between 1991 and 2012, plotted by calendar year, year of birth, and age, respectively (Figure 1). As can be seen from the graphs, patients born between 1951 and 1964 had the highest HIV prevalence (about 25%), and HIV prevalence increased until the age of 35 years and then stabilized. However, all groups of non-injectors had a four to five times lower RR of HIV prevalence as the reference group of Swiss males who ever injected (Table 3). A significantly higher risk ratio of HIV prevalence appeared among ever-injecting females in the complete dataset, as well as in the imputed dataset (Swiss RR 1.18, CI 95% 1.04–1.34; non-Swiss RR 1.58, CI 95% 1.18–2.12). Finally, the declining trend in HIV prevalence during the calendar year was equal for all subgroups, regardless of gender, nationality, or lifetime injecting status (Figure 2).

**Table 2:** Model estimates of predictors of HIV-positive status according to GEE analysis

**Independent variables**	**Complete dataset**			**Imputed dataset**		
	**Estimate**	**SE**	***P *** **value**	**Estimate**	**SE**	***P *** **value**
Intercept	-0.593	0.050	<0.001	-0.631	0.048	<0.001
Calendar year	-0.277	0.038	<0.001	-0.254	0.036	<0.001
Year of birth	-0.039	0.009	<0.001	-0.043	0.009	<0.001
Year of birth^a^	-0.054	0.012	<0.001	-0.042	0.010	<0.001
Year of birth^b^	0.019	0.005	<0.001	0.013	0.005	0.012
Age	0.043	0.009	<0.001	0.035	0.008	<0.001
Age^c^	-0.048	0.006	<0.001	-0.043	0.006	<0.001
Age^d^	0.012	0.002	<0.001	0.011	0.002	<0.001
Female, non-Swiss, non-injector	-1.760	0.492	<0.001	-1.435	0.543	0.011
Female, non-Swiss, ever injector	0.497	0.158	0.002	0.457	0.150	0.002
Female, Swiss, non-injector	-1.344	0.197	<0.001	-1.372	0.201	<0.001
Female, Swiss, ever injector	0.186	0.069	0.007	0.167	0.065	0.011
Male, non-Swiss, non-injector	-1.520	0.299	<0.001	-1.422	0.261	<0.001
Male, non-Swiss, ever injector	-0.074	0.109	0.496	-0.087	0.107	0.417
Male, Swiss, non-injector	-1.547	0.149	<0.001	-1.567	0.143	<0.001

The time variables were rescaled to fit the GEE model as follows: calendar year = logarithm of year - 1990, year of birth = year - 1960, age = age - 30. ^a^Year of birth = Year of birth × Year of birth / 10. ^b^Year of birth = Year of birth × Year of birth × Year of birth / 100. ^c^Age = Age × Age / 10, ^d^Age = Age × Age × Age / 100. The group ‘Male, Swiss, ever injector’ was reference category and is therefore omitted from the independent variable list. *SE*, standard error.

**Table 3:** Risk ratio (RR) of predictors of HIV-positive status according to GEE analysis

**Independent variables**	**Complete dataset**			**Imputed dataset**		
	**RR**	**CI 95%-**	**CI 95%+**	**RR**	**CI 95%-**	**CI 95%+**
Calendar year	0.76	0.70	0.82	0.78	0.72	0.83
Year of birth	0.96	0.94	0.98	0.96	0.94	0.97
Year of birth^a^	0.95	0.93	0.97	0.96	0.94	0.98
Year of birth^b^	1.02	1.01	1.03	1.01	1.00	1.02
Age	1.04	1.03	1.06	1.04	1.02	1.05
Age^c^	0.95	0.94	0.96	0.96	0.95	0.97
Age^d^	1.01	1.01	1.02	1.01	1.01	1.02
Female, non-Swiss, non-injector	0.17	0.07	0.45	0.24	0.08	0.69
Female, non-Swiss, ever injector	1.64	1.21	2.24	1.58	1.18	2.12
Female, Swiss, non-injector	0.26	0.18	0.38	0.25	0.17	0.38
Female, Swiss, ever injector	1.20	1.05	1.38	1.18	1.04	1.34
Male, non-Swiss, non-injector	0.22	0.12	0.39	0.24	0.14	0.40
Male, non-Swiss, ever injector	0.93	0.75	1.15	0.92	0.74	1.13
Male, Swiss, non-injector	0.21	0.16	0.28	0.21	0.16	0.28

The time variables were rescaled to fit the GEE model as follows: calendar year = logarithm of year - 1990, year of birth = year - 1960, age = age - 30. ^a^Year of birth = Year of birth × Year of birth / 10. ^b^Year of birth = Year of birth × Year of birth × Year of birth / 100. ^c^Age = Age × Age / 10, ^d^Age = Age × Age × Age / 100. The group ‘Male, Swiss, ever injector’ was reference category and is therefore omitted from the independent variable list.

## Discussion

In analyzing this case register, we aimed to identify ethnic- and gender-specific differences in the prevalence of HIV among heroin users receiving opioid maintenance treatment in the canton of Zurich, Switzerland. Overall, we found a significant decrease in the prevalence of HIV among opioid-maintained patients, regardless of gender and nationality, during the first years after initiation of the register in 1991. The following years were associated with a more stable prevalence of HIV in this subgroup.

These findings are in line with previously available data from the Swiss Federal Office of Public Health and the SWISS HIV Cohort Study [19,24,26] and underline the effectiveness of measures that have been taken since HIV/AIDS became a major public health concern in the context of open drug scenes that developed in Switzerland’s largest cities in the early 1980s. Between 1985 and 1995, HIV/AIDS incidence and prevalence rates were the highest in Europe [56,57]. Virus transmission by contaminated syringes and needles among an estimated 3,000 IDUs contributed significantly to this crisis [57]. Although methadone prescription and maintenance treatment had been introduced almost a decade earlier and all physicians could prescribe methadone until 1975, controlled-substance legislation was inconsistent and oscillated between liberal and restrictive approaches [21]. In the face of this heroin and HIV/AIDS epidemic, a harm-reduction-friendly policy was adopted in 1991 at the federal level [21,57], which formally permitted the implementation of low-threshold methadone programs, needle- and syringe-exchange services, supervised consumption rooms, and heroin-assisted treatments [58].

When this legislation was implemented in cantonal practice in 1991, administrative barriers in Zurich (and other Swiss cities) were greatly reduced [59], since it was recognized that high-entry barriers keep opiate-dependent users from seeking admission to OMT. Since then, this finding has been widely and repeatedly replicated [60-63]. Furthermore, studies from Canada show that limited provision of addiction treatment may result not just in a missed opportunity to reduce HIV transmission behavior among IDUs [64] but could lead to increased HIV-related expenditures for the general public [65].

Other than suffering from an opiate dependence, there were no further prerequisites to enter treatment. Furthermore, medical care and prescription of opioids such as methadone and buprenorphine were covered by mandatory health insurance, and sufficient OMT providers were involved to avoid waiting lists. The introduction of an on-call service, staffed 24/7, allowed the admission of opiate-dependent patients into an OMT and the dispensation of a first dosage of methadone without delay. Both cantonal and private treatment institutions offered multidisciplinary approaches and were usually staffed by psychiatrists and internal medicine specialists with nurses, as well as social workers, to provide widespread support [21,57]. Special attention was given to pregnant women suffering from opioid dependence to ensure easy access to OMT during pregnancy and after childbirth [66].

It was estimated that OMT attracts around 64% of Zurich’s opioid-dependent patients [51]. Furthermore, every second individual with a problematic use of opioids will, regardless of gender, seek admission into a program within 2–3 years after developing dependency [51].

Despite this low-threshold approach, our findings suggest that gender and migration background influence the risk of being HIV positive. Different access to treatment and different exposure risk characteristics for certain subgroups may offer possible explanations for these findings [67-69]. A recent review identified pregnancy, lack of services for pregnant women, fear of losing custody for a newborn, or fear of prosecution, coupled with lack of childcare outside of treatment, to be gender-specific barriers keeping women from entering treatment for substance use disorders in general [70-72]. Additionally, women may not just lack social support, but experience greater social stigma and discrimination than their male counterparts, when entering such treatment [70,73,74]. Women have also been reported to articulate more negative expectations about treatment than males [70,75]. A number of studies documented systematic barriers—irrespective of gender—related to the policies and procedures of OMT [76]. They include multiple requirements for treatment initiation or modification (including waiting lists), rules regarding abstinence, requirements of established health insurance, hidden or collateral fees, requirements to prove identification, limited take-home dose availability, and a lack of information regarding treatment options [77,78]. One aspect may be that drug-related services in Switzerland do not tend to be culture-specific, so as not to specifically arrange services for certain migrant subgroups, which may have the effect of increasing the likelihood of cultural misperception and overlook social variables. Previous studies elsewhere showed that migrants without legal documentation might avoid seeking medical advice or entering treatment services because of fear of expulsion from the country [27,79]. Furthermore, some authors reported a more serious progression of opioid dependence in an immigrant population (leading to the acquisition of multiple infections) and interpreted this finding as part of a multifaceted acculturation problem [80].

In addition, the higher risk for native women and for women with a migration background warrants further research and should identify what factors deter women from using available HIV-prevention measures, as well as whether these measures need to be better adapted to high-risk groups. This becomes even more apparent in light of a recent study among a Canadian cohort of HIV-positive individuals in IDUs, which identified female gender as an additional barrier to access and adherence to antiretroviral therapy once women had been infected—a finding that was independent of drug use and other socio-behavioral and clinical characteristics [81].

We acknowledge the limitations of our study. First, Switzerland’s immigration policy is strictly controlled, especially in relation to individuals from non-EU member states and relies on a system of quotas and permits (short-term permit (L), initial residence permit (B), permanent residence permit (C), and cross border commuter permits (G)) and may prevent naturalization for two or more generations [82]. Our calculations might therefore have falsely categorized individuals as non-native who were actually born and raised in Switzerland (i.e., granting nationality on the basis of *jus sanguinis* (‘right of blood’), as compared to those granting it on the grounds of *jus soli* (‘right of soil’), such as the USA.

Second, because of our small sample size, we cannot provide a finer breakdown for analysis than Swiss and non-Swiss; therefore, the latter is an extremely heterogeneous group that includes individuals from the majority of European and Eastern European countries, as well as a few subjects of Arabic or African ethnic background. Nevertheless, our database of OMT is one of the oldest in existence and is the largest of its kind in Europe, so our findings might still be useful when comparing HIV prevalence rates internationally, especially in relation to gender and immigration background.

## Conclusions

These findings provide insights into ethnic- and gender-specific differences in the prevalence of HIV among heroin users receiving opioid maintenance treatment in the canton of Zurich, Switzerland. In this sample, overall prevalence rates of HIV decreased significantly since 1991, which can be associated with the introduction of widespread harm-reduction measures. In particular, the higher risk for women who inject drugs, and especially for women with a migrant background, warrants additional research and needs to address what factors deter this subgroup from using available HIV-prevention measures.

## Competing interests

The authors declare that they have no competing interests.

## Authors’ contributions

CN was the main contributor to the design of the study and did the statistical analyses. ML drafted the manuscript and was supported by RS and CN. All authors read and approved the final manuscript.

## References

[B1] Bruce RD Methadone as HIV prevention: high volume methadone sites to decrease HIV incidence rates in resource limited settings Int J Drug Policy 2009 21 2 122 124 19931444 10.1016/j.drugpo.2009.10.004 PMC2839048

[B2] Brugal MT Domingo-Salvany A Puig R Barrio G Garcia De Olalla P De La Fuente L Evaluating the impact of methadone maintenance programmes on mortality due to overdose and aids in a cohort of heroin users in Spain Addiction 2005 100 981 989 15955014 10.1111/j.1360-0443.2005.01089.x

[B3] Gowing L Farrell M Bornemann R Sullivan L Ali R Substitution treatment of injecting opioid users for prevention of HIV infection Cochrane Database Syst Rev 2008 2 CD004145 18425898 10.1002/14651858.CD004145.pub3

[B4] van den Brink W van Ree JM Pharmacological treatments for heroin and cocaine addiction Eur Neuropsychopharmacol 2003 13 476 487 14636964 10.1016/j.euroneuro.2003.08.008

[B5] Reddon H Milloy MJ Simo A Montaner J Wood E Kerr T Methadone maintenance therapy decreases the rate of antiretroviral therapy discontinuation among HIV-positive illicit drug users AIDS Behav 2013 18 4 740 746 23918244 10.1007/s10461-013-0584-z PMC4059183

[B6] Metzger DS Woody GE O’Brien CP Drug treatment as HIV prevention: a research update J Acquir Immune Defic Syndr 2010 55 Suppl 1 S32 S36 21045597 10.1097/QAI.0b013e3181f9c10b PMC3155766

[B7] Lawrinson P Ali R Buavirat A Chiamwongpaet S Dvoryak S Habrat B Jie S Mardiati R Mokri A Moskalewicz J Newcombe D Poznyak V Subata E Uchtenhagen A Utami DS Vial R Zhao C Key findings from the WHO collaborative study on substitution therapy for opioid dependence and HIV/AIDS Addiction 2008 103 1484 1492 18636999 10.1111/j.1360-0443.2008.02249.x

[B8] Caplehorn JR Ross MW Methadone maintenance and the likelihood of risky needle-sharing Int J Addict 1995 30 685 698 7657397 10.3109/10826089509048753

[B9] Drucker E Lurie P Wodak A Alcabes P Measuring harm reduction: the effects of needle and syringe exchange programs and methadone maintenance on the ecology of HIV AIDS 1998 12 Suppl A S217 S230 9633006

[B10] Ball JC Lange WR Myers CP Friedman SR Reducing the risk of AIDS through methadone maintenance treatment J Health Soc Behav 1988 29 214 226 3241064

[B11] Newman RG Intravenous drug use and HIV Lancet 2006 368 1574 17084756 10.1016/S0140-6736(06)69661-X

[B12] Calsyn DA Crits-Christoph P Hatch-Maillette MA Doyle SR Song YS Coyer S Pelta S Reducing sex under the influence of drugs or alcohol for patients in substance abuse treatment Addiction 2010 105 100 108 20078464 10.1111/j.1360-0443.2009.02812.x PMC2808629

[B13] Woody GE VanEtten-Lee ML McKirnan D Donnell D Metzger D Seage G 3rd Gross M Substance use among men who have sex with men: comparison with a national household survey J Acquir Immune Defic Syndr 2001 27 86 90 11404525 10.1097/00126334-200105010-00015

[B14] Tross S Hanner J Hu MC Pavlicova M Campbell A Nunes EV Substance use and high risk sexual behaviors among women in psychosocial outpatient and methadone maintenance treatment programs Am J Drug Alcohol Abuse 2009 35 368 374 20180666 10.1080/00952990903108256 PMC2846384

[B15] Bellis DJ Reduction of AIDS risk among 41 heroin addicted female street prostitutes: effects of free methadone maintenance J Addict Dis 1993 12 7 23 8381030 10.1300/J069v12n01_02

[B16] Zenker C First results of a methadone programme for drug-addicted women prostituting themselves Eur Addict Res 1995 1 139 145

[B17] Kerr T Wodak A Elliott R Montaner JS Wood E Opioid substitution and HIV/AIDS treatment and prevention Lancet 2004 364 1918 1919 15566992 10.1016/S0140-6736(04)17490-4

[B18] Herget G Methadone and buprenorphine added to the WHO list of essential medicines HIV AIDS Policy Law Rev 2005 10 23 24 16544403

[B19] Pluess L Frey K Kuebler D Rosenbrock R Review of the Swiss HIV Policy by a Panel of International Experts - A Study on Behalf of the Swiss Federal Office of Public Health 2009 Syntagma GmbH 73

[B20] de Jong W Weber U The professional acceptance of drug use: a closer look at drug consumption rooms in the Netherlands, Germany and Switzerland Int J Drug Policy 1999 10 99 108

[B21] Klingemann HK Drug treatment in Switzerland: harm reduction, decentralization and community response Addiction 1996 91 723 736 8935256 10.1046/j.1360-0443.1996.9157238.x

[B22] Nelles J Stoever H Zehn jahre spritzenvergabe im gefaengnis: ein review der bisherigen spritzenvergabeprojekte in der Schweiz, Deutschland, Spanien und Moldawien Suchttherapie 2002 3 155 161

[B23] Dubois-Arber F Balthasar H Huissoud T Zobel F Arnaud S Samitca S Jeannin A Schnoz D Gervasoni JP Trends in drug consumption and risk of transmission of HIV and hepatitis C virus among injecting drug users in Switzerland, 1993–2006 Euro Surveill 2008 13 21 717 727 10.2807/ese.13.21.18881-en 18761964

[B24] Benninghoff F Morency P Geense R Huissoud T Dubois-Arber F Health trends among drug users attending needle exchange programmes in Switzerland (1994–2000) AIDS Care 2006 18 371 375 16809115 10.1080/09540120500429018

[B25] Sudre P Rickenbach M Taffe P Janin P Volkart AC Francioli P Clinical epidemiology and research on HIV infection in Switzerland: the Swiss HIV Cohort Study 1988–2000 Schweiz Med Wochenschr 2000 130 1493 1500 11075414

[B26] Schoeni-Affolter F Ledergerber B Rickenbach M Rudin C Gunthard HF Telenti A Furrer H Yerly S Francioli P Cohort profile: the Swiss HIV Cohort study Int J Epidemiol 2010 39 1179 1189 19948780 10.1093/ije/dyp321

[B27] Rachlis B Brouwer KC Mills EJ Hayes M Kerr T Hogg RS Migration and transmission of blood-borne infections among injection drug users: understanding the epidemiologic bridge Drug Alcohol Depend 2007 90 107 119 17485179 10.1016/j.drugalcdep.2007.03.014

[B28] Johnson TP VanGeest JB Cho YI Migration and substance use: evidence from the U.S. National Health Interview Survey Subst Use Misuse 2002 37 941 972 12180572 10.1081/ja-120004160

[B29] Freeman RC Williams ML Saunders LA Drug use, AIDS knowledge, and HIV risk behaviors of Cuban-, Mexican-, and Puerto-Rican-born drug injectors who are recent entrants into the United States Subst Use Misuse 1999 34 1765 1793 10540972 10.3109/10826089909039426

[B30] Paschane DM Fisher DG Etiology of limited transmission diseases among drug users: does recent migration magnify the risk of sharing injection equipment? Soc Sci Med 2000 50 1091 1097 10714929 10.1016/s0277-9536(99)00357-3

[B31] Barry D Weinstock J Petry NM Ethnic differences in HIV risk behaviors among methadone-maintained women receiving contingency management for cocaine use disorders Drug Alcohol Depend 2008 98 144 153 18684571 10.1016/j.drugalcdep.2008.06.009 PMC2614896

[B32] Denner J Organista KC Dupree JD Thrush G Predictors of HIV transmission among migrant and marginally housed Latinos AIDS Behav 2005 9 201 210 15933839 10.1007/s10461-005-3901-3

[B33] Rekart ML Sex-work harm reduction Lancet 2005 366 2123 2134 16360791 10.1016/S0140-6736(05)67732-X

[B34] Shannon K Kerr T Allinott S Chettiar J Shoveller J Tyndall MW Social and structural violence and power relations in mitigating HIV risk of drug-using women in survival sex work Soc Sci Med 2008 66 911 921 18155336 10.1016/j.socscimed.2007.11.008

[B35] Shannon K Kerr T Bright V Gibson K Tyndall M Drug sharing with clients as a risk marker for increased violence and sexual and drug-related harms among survival sex workers AIDS Care 2008 20 228 234 18293134 10.1080/09540120701561270

[B36] Goodey J Migration, crime and victimhood responses to sex trafficking in the EU Punishment Soc 2003 5 415 431

[B37] Schilling RF El Bassel N Schinke SP Nichols S Botvin GJ Orlandi MA Sexual behavior, attitudes toward safer sex, and gender among a cohort of 244 recovering i.v. drug users Int J Addictions 1991 26 859 877 10.3109/10826089109058926 1960004

[B38] Harrison DF Wambach KG Byers JB Imershein AW Levine P Maddox K Quadagno DM Fordyce ML Jones MA AIDS knowledge and risk behaviors among culturally diverse women AIDS Educ Prev 1991 3 79 89 1873140

[B39] Grella CE Annon JJ Anglin MD Ethnic differences in HIV risk behaviors, self-perceptions, and treatment outcomes among women in methadone maintenance treatment J Psychoactive Drugs 1995 27 421 433 8788697 10.1080/02791072.1995.10471706

[B40] Gross DM Immigration Policy and Foreign Population in Switzerland 2006 Simon Fraser University, Vancouver BC, Canada: World Bank

[B41] Riano Y Wastl-Walter D Immigration policies, state discourses on foreigners, and the politics of identity in Switzerland Environ Plann 2006 38 1693

[B42] Straubhaar T Weber R On the economics of immigration: some empirical evidence for Switzerland Int Rev Appl Econ 1994 8 107 129

[B43] Fuer Migration B Migrationsbericht 2011 2012 Bern Wabern: Bundesamt fuer Migration

[B44] Longchamp C Sans Papiers in der Schweiz: Arbeitsmarkt, nicht Asylpolitik ist entscheidend: Schlussbericht im Auftrag des Bundesamtes fuer Migration 2005 GfS-Forschungsinstitut, Politik und Staat

[B45] Theurl E Some aspects of the reform of the health care systems in Austria, Germany and Switzerland Health Care Anal 1999 7 331 354 10787796 10.1023/A:1009426731833

[B46] Schellhorn M The effect of variable health insurance deductibles on the demand for physician visits Health Econ 2001 10 441 456 11466805 10.1002/hec.630

[B47] Colombo F Towards More Choice in Social Protection?: Individual Choice of Insurer in Basic Mandatory Health Insurance in Switzerland 2001 Paris: OECD Publishing

[B48] Johann A Breitenmoser S Uebersax P Legalitaet in der Illegalitaet: Die migrationsrechtliche Stellung von Papierlosen (Sans-Papiers)

[B49] Bestand der ständingen ausländischen Wohnbevölkerung nach Wohnkanton und Ausländergruppe [https://www.bfm.admin.ch/content/dam/data/migration/statistik/auslaenderstatistik/2013/auslaenderstatistik-2013-08-d.pdf]

[B50] Falcato L Stohler R Dursteler-Mac Farland KM Eichenberger A Eich D Rossler W Closure of an open drug scene–a case register-based analysis of the impact on the demand for methadone maintenance treatment Addiction 2001 96 623 628 11300965 10.1046/j.1360-0443.2001.96462310.x

[B51] Nordt C Stohler R Incidence of heroin use in Zurich, Switzerland: a treatment case register analysis Lancet 2006 367 1830 1834 16753485 10.1016/S0140-6736(06)68804-1

[B52] Fingerhuth T Tschurr C Betaubungsmittelgesetz: das BetmG und weitere Bundesgesetze (in Auszuegen), Verordnungen sowie voelkerrechtliche Vertraege mit Verweisungen, Anmerkungen, Hinweisen und Sachregister: [Kommentar]. orell fuessli 2007

[B53] Uchtenhagen A Dobler-Mikola A Gutzwiller F Medical prescription of narcotics Eur Addict Res 1996 2 201 207

[B54] Zou GY Donner A Extension of the modified Poisson regression model to prospective studies with correlated binary data Stat Methods Med Res 2013 22 661 670 22072596 10.1177/0962280211427759

[B55] Liang K-Y Zeger SL Longitudinal data analysis using generalized linear models Biometrika 1986 73 13 22

[B56] Dubois-Arber F Jeannin A Spencer B Long term global evaluation of a national AIDS prevention strategy: the case of Switzerland AIDS 1999 13 2571 2582 10630527 10.1097/00002030-199912240-00011

[B57] Uchtenhagen A Heroin-assisted treatment in Switzerland: a case study in policy change Addiction 2010 105 29 37 19922519 10.1111/j.1360-0443.2009.02741.x

[B58] Uchtenhagen A The medical prescription of heroin to heroin addicts Drug Alcohol Rev 1997 16 297 298 16203439 10.1080/09595239800187481

[B59] Haemmig RB Harm reduction in Bern: from outreach to heroin maintenance Bull N Y Acad Med 1995 72 371 379 10101377 PMC2359428

[B60] Lin C Wu Z Detels R Opiate users’ perceived barriers against attending methadone maintenance therapy: a qualitative study in China Subst Use Misuse 2011 46 9 1190 1198 21417558 10.3109/10826084.2011.561905 PMC3392119

[B61] Hyshka E Strathdee S Wood E Kerr T Needle exchange and the HIV epidemic in Vancouver: lessons learned from 15 years of research Int J Drug Policy 2012 23 261 270 22579215 10.1016/j.drugpo.2012.03.006 PMC3392518

[B62] Van Den Berg C Smit C Van Brussel G Coutinho R Prins M Full participation in harm reduction programmes is associated with decreased risk for human immunodeficiency virus and hepatitis C virus: evidence from the Amsterdam Cohort Studies among drug users Addiction 2007 102 1454 1462 17697278 10.1111/j.1360-0443.2007.01912.x PMC2040242

[B63] Wiessing L Likatavicius G Klempova D Hedrich D Nardone A Griffiths P Associations between availability and coverage of HIV-prevention measures and subsequent incidence of diagnosed HIV infection among injection drug users Am J Public Health 2009 99 1049 1052 19372511 10.2105/AJPH.2008.141846 PMC2679784

[B64] Wood E Spittal P Li K Kerr T Miller CL Hogg RS Montaner JS Schechter MT Inability to access addiction treatment and risk of HIV infection among injection drug users J Acquir Immune Defic Syndr 2004 36 750 754 15167295 10.1097/00126334-200406010-00013

[B65] Kuyper LM Hogg RS Montaner JS Schechter MT Wood E The cost of inaction on HIV transmission among injection drug users and the potential for effective interventions J Urban Health 2004 81 655 660 15466846 10.1093/jurban/jth148 PMC3455935

[B66] Kashiwagi M Arlettaz R Lauper U Zimmermann R Hebisch G Methadone maintenance program in a Swiss perinatal center: (I): management and outcome of 89 pregnancies Acta Obstet Gynecol Scand 2005 84 140 144 15683373 10.1111/j.0001-6349.2005.00497.x

[B67] Squires KE Gender differences in the diagnosis and treatment of HIV Gend Med 2007 4 294 307 18215722 10.1016/s1550-8579(07)80060-x

[B68] Spittal PM Craib KJ Wood E Laliberte N Li K Tyndall MW O’Shaughnessy MV Schechter MT Risk factors for elevated HIV incidence rates among female injection drug users in Vancouver CMAJ 2002 166 894 899 11949985 PMC100922

[B69] Staehelin C Rickenbach M Low N Egger M Ledergerber B Hirschel B D’Acremont V Battegay M Wagels T Bernasconi E Kopp C Furrer H Swiss HIV Cohort Study Migrants from Sub-Saharan Africa in the Swiss HIV Cohort Study: access to antiretroviral therapy, disease progression and survival AIDS 2003 17 2237 2244 14523281 10.1097/00002030-200310170-00012

[B70] Greenfield SF Brooks AJ Gordon SM Green CA Kropp F McHugh RK Lincoln M Hien D Miele GM Substance abuse treatment entry, retention, and outcome in women: a review of the literature Drug Alcohol Depend 2007 86 1 21 16759822 10.1016/j.drugalcdep.2006.05.012 PMC3532875

[B71] Schober R Annis HM Barriers to help-seeking for change in drinking: a gender-focused review of the literature Addict Behav 1996 21 81 92 8729710 10.1016/0306-4603(95)00041-0

[B72] Ashley OS Marsden ME Brady TM Effectiveness of substance abuse treatment programming for women: a review Am J Drug Alcohol Abuse 2003 29 19 53 12731680 10.1081/ada-120018838

[B73] Neale J Tompkins C Sheard L Barriers to accessing generic health and social care services: a qualitative study of injecting drug users Health Soc Care Commun 2008 16 147 154 10.1111/j.1365-2524.2007.00739.x 18290980

[B74] Finkelstein N Treatment issues for alcohol-and drug-dependent pregnant and parenting women Health Soc Work 1994 19 7 15 8168782 10.1093/hsw/19.1.7

[B75] Kline A Pathways into drug user treatment: the influence of gender and racial/ethnic identity Subst Use Misuse 1996 31 323 342 8834265 10.3109/10826089609045815

[B76] Wolfe D Carrieri MP Shepard D Treatment and care for injecting drug users with HIV infection: a review of barriers and ways forward Lancet 2010 376 355 366 20650513 10.1016/S0140-6736(10)60832-X

[B77] Mattick RP Kimber J Breen C Davoli M Buprenorphine maintenance versus placebo or methadone maintenance for opioid dependence Cochrane Database Syst Rev 2014 2 61 10.1002/14651858.CD002207.pub4 PMC10617756 24500948

[B78] Blankertz L Magura S Staines GL Madison EM Spinelli M Horowitz E Bali P Guarino H Grandy A Young R A new work placement model for unemployed methadone maintenance patients Subst Use Misuse 2004 39 2239 2260 15603003 10.1081/ja-200034600

[B79] Apostolopoulos Y Sonmez S Kronenfeld J Castillo E McLendon L Smith D STI/HIV risks for Mexican migrant laborers: exploratory ethnographies J Immigr Minor Health 2006 8 291 302 16791539 10.1007/s10903-006-9334-2

[B80] Reimer J Lorenzen J Baetz B Fischer B Rehm J Backmund M Haasen C Injection drug use, multiple hepatitis virus infections, and migration: a German study Subst Use Misuse 2007 42 1353 1365 17886136 10.1080/10826080701205760

[B81] Tapp C Milloy MJ Kerr T Zhang R Guillemi S Hogg RS Montaner J Wood E Female gender predicts lower access and adherence to antiretroviral therapy in a setting of free healthcare BMC Infect Dis 2011 11 86 21466704 10.1186/1471-2334-11-86 PMC3080305

[B82] Fischer A Nicolet S Sciarini P Europeanisation of a Non-EU Country: the Case of Swiss Immigration Policy West Eur Politics 2002 25 143 170

